# The Hog Bacon, Battles and By-Gone Days

**Published:** 1889-01

**Authors:** Daniel Parker

**Affiliations:** Calvert, Texas


### CORRESPONDENCE.

### THE HOG BACON, BATTLES AND BY-GONE DAYS.

LETTER FROM DR. DANIEL PARKER.

*Editor Daniel's Texas Medical Journal:*

I had hoped that a sober second thought on the hog question would have produced in your heart profound symptoms of repentance if not of remorse, but I am grieved to see that your last issue, notwithstanding the lucid letter from Dr. Cunningham, contains evidence of contumaciousness that cannot be ignored, and renders it necessary that some one of your many friends should labor with you still further.

If it were a question as to whether the decayed viscera or poisoned flesh of the hog are suitable for food, or whether they are better or worse than like parts in like condition of "lambs without blemish of the first year," or any "clean" animal, I would have nothing to say; but the product of the healthy hog is too important a factor in our economy, too essential as a heat and force producer to be discarded because some wretch has sought to make food of his guts and poisoned flesh.

The mast-fed "razor-back" has always been in the van-guard of civilization in its westward march, and his vigorous squeal, startling the Sylvan deities, has proclaimed that their reign was at an end, and that the advent of the ax and plow was at hand; while the sputter of the frying-pan, as his flesh was being nicely browned, has made sweet music in many a frontier cabin. No doubt Moses understood that his people were to take possession of the land of the Hittites, Jebusites, et al., with their fields and vineyards—no wildernesses to be subdued, nor bushes to grub up—when he forebade swine flesh.

And again, the more favored brothers of the "razor-back," the improved hog plays a no less important role in the development of all our material resources. Prof. Atwater * tells us that the heat or force producing capacities of fat pork, beef and mutton represented in calorics, are respectively: 3452, 2750 and 1906. "Hog and hominy" is in front! How could we clear our forests, till our lands, build our railroads, work our mines, or figh our battles without bacon?

This brings to mind visions of the past, which will doub ess stir a responsive chord in your breast, and certainly will in the breasts of many of your readers who followed the fortunes of "the Lost Cause." At the close of a days hard march, and on the eve of battle, did you never drop down by your camp-fire, ransack your haversack for your pittance of bacon—the only ration capable of withstanding the adverse inflnence of heat, cold and rain—and while your tin cup of parched-meal-coffee was simmering on the coals, suspend it over the flames, pierced by your ramrod, and as the drops of grease would fall catch them on your "pone" of unbolted corn-meal bread? Many have realized this picture:

O! how our salivary glands (sublingual and parotid),
Overflowed, as in our hands the "pone" with precious drops was
dotted!
How fragrant was the smoky air, like incense heavenward rising,
And as we sniffed the od'rous breeze, we felt our hopes reviving!
For in that ration adispose, lay force, and red corpuseles,
To fill our veins, and to renew our weary wasted muscles!
No fears of noxious *tenia solium*, nor of *eechino coccus*,
Trichina S. nor bacilli, nor deadly urinococcus,
Disturbed our gustatory joys, nor hungry visions haunted,
Bugs, germs and all, we bolted down, by hidden foes undaunted;
Our scanty meal too soon consumed, lights out, our drummers
rattled,
While distant, growling, hostile guns, presaged to-morrow's
battle;
And when at last our weary frames by sleep were overtaken,
Our fitful dreams were, half and half, of *victory* and *bacon!*

---

* Century Magazine, July, 1887.

Don't ask those of us who have fried bacon on our ramrods, to join in a crusade against swines fat.

So much, in regard to the hog as *meat*. As a *hog* he is able to take care of himself. The best is always good enough for him. He eats the best food and sleeps in the best bed he can find. If these are good, he thrives ; if they are unwholesome and dirty, he still survives; thus showing a wide range of adaptability, and indicating that in a race for "survival of the fitest," he has good staying qualities

The day may come when mankind will be educated up to the point of eshewing his flesh, but if so, it will be a thousand years after they have quit "cussin' and chawin' tobacker," and when the record of the last battle will be more ancient than the Sinaitic manuscript now is. For the present we "need him in our business."

Yours respectfully,

DANIEL PARKER, M. D.

CALVERT, TEXAS, Jan. 7, 1889.

[The doctor has, indeed, "touched a tender chord" in our memory. "We 'uns" have often realized the details of the camp picture he has so touchingly drawn, and, if anything could reconcile us to the use of hog's flesh as food, it would be the grateful remembrance of how it served us "'en during' of the war"—as Johny Reb' used to serve it up—*a la* ramrod. We make our bow to Dr. Parker, and acknowledge the strength, the incontrovertibility of his argument, and we will acknowledge that hogs flesh is one of the sinews of war and of labor ; but in doing so we do not acknowledge that it is indispensable. There are many "heat and force" makers to be found, free from the objections which attach to the hog's flesh, and which will, doubtless, some day take its place.

If, for no other reason than to have drawn out the above ex cellent article, with its touching "pome,,' and the sensible letter of Dr. Cunningham, we are glad we wrote the "attack on the hog." Let us hear from others of our readers on the subject.—ED.]

